# Mitigating Freezing Artifacts in Polar Coarse-Grained Water through Rebalancing Electrostatic and van der Waals Interactions: A Case Study of the pSPICA Force Field

**DOI:** 10.1021/acs.jpcb.6c06050

**Published:** 2026-10-08

**Authors:** Yi-Chen Tsai, Qun-Yan Cheng, Wataru Shinoda, Chi-Cheng Chiu

**Affiliations:** † Department of Chemical Engineering, 34912 National Cheng Kung University, Tainan 701, Taiwan; ‡ Research Institute for Interdisciplinary Science, Okayama University, Okayama 700-8530, Japan; § Center for Resilience and Intelligence on Sustainable Energy Research (RiSER), National Cheng Kung University, Tainan 701, Taiwan; ∥ Program on Smart and Sustainable Manufacturing, Academy of Innovative Semiconductor and Sustainable Manufacturing, National Cheng Kung University, Tainan 701, Taiwan; ⊥ Fire Protection and Safety Research Center, National Cheng Kung University, Tainan 711, Taiwan

## Abstract

Coarse-grained (CG) water models with explicit electrostatic interactions are essential for capturing the dielectric response of biomolecular systems. However, many CG water models exhibit pronounced freezing artifacts under ambient conditions. Using the SPICA force-field family as a case study, we examined the influences of charge topology, background dielectric screening (ε_bg_), and Lennard–Jones (LJ) interaction strength on the freezing tendency of CG water. Among the models examined here, which all reproduce the target density and surface tension at 298 K, changing the charge topology from a linear dipole to V-shaped geometries did not substantially lower the melting temperature (*T*_m_). Instead, *T*_m_ was more sensitive to the LJ interaction strength. Reducing ε_bg_ strengthens the electrostatic contribution, allowing the LJ attraction to be weakened while maintaining the target properties. On this basis, we propose a parametrization framework for introducing a permanent dipole into a nonpolar CG water model while mitigating its freezing tendency. Using this framework, we developed pSPICA2, which reduces *T*_m_ from 384 to 302 K while retaining accurate density, surface tension, and dielectric response at 298 K. Although *T*_m_ remains above the experimental value of 273 K in this work, the proposed framework offers a practical route for developing polar CG water models with reduced freezing artifacts.

## Graphical abstract

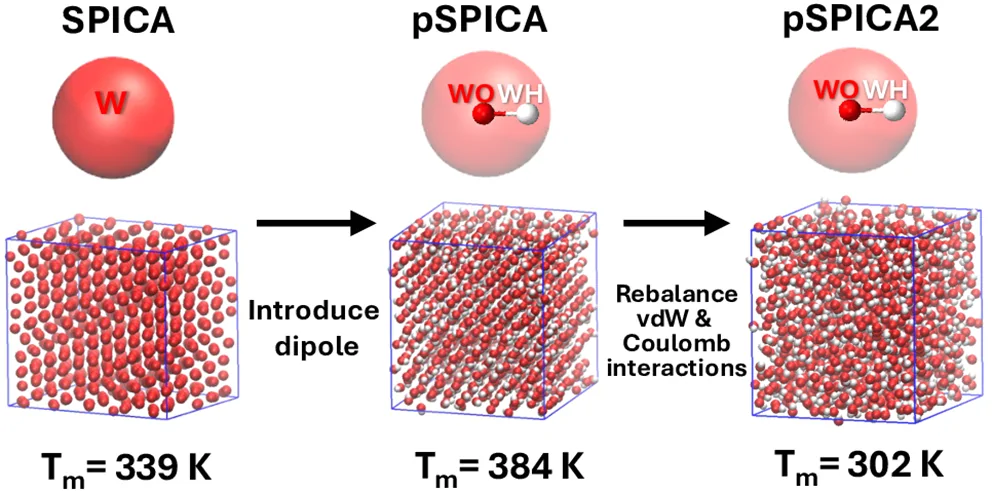

## Introduction

Molecular dynamics (MD) simulations have become an important tool for investigating the structural, dynamical, and thermodynamic behavior of biological systems and soft-matter assemblies. Despite substantial advances in computer hardware and simulation algorithms, all-atom (AA) MD simulations remain fundamentally constrained in the time and length scales that they can access. This limitation restricts their applicability to many biologically relevant processes that occur on microsecond or longer time scales or involve mesoscale structural reorganization. Coarse-grained (CG) force fields (FFs) address this limitation by reducing the number of degrees of freedom, thereby extending accessible simulation time and length scales while retaining essential physicochemical features of the underlying molecular interactions.^1–5^

Among the CG FFs developed for biomolecular simulations, the MARTINI FF has become one of the most widely used frameworks because of its systematic parametrization based on partitioning free energies and its broad applicability to lipids, proteins, and other biomolecules.^3,6–11^ Its applications now extend well beyond lipid membranes to proteins, carbohydrates, and intrinsically disordered proteins, reflecting the continuing refinement and growing versatility of the framework.^7,9,12^ Independently, the SPICA FF (formerly the SDK FF) emphasizes the reproduction of experimental interfacial tensions and densities, together with distribution functions derived from AA-MD reference simulations.^13–20^ The SPICA FF has demonstrated strong performance in describing domain formation and the mesoscale morphology of lipid assemblies.^17,21,22^

Because water mediates nearly all intermolecular interactions relevant to the structures and dynamics in biological assemblies, the fidelity of the water representation directly constrains the range of phenomena a CG FF can capture.^23–28^ Accordingly, CG water models have been developed with different target properties for different CG FF frameworks. In their standard forms, both MARTINI and SPICA represent water as a single uncharged particle and use an implicit background dielectric constant to approximate electrostatic screening.^6,13^ The two frameworks, however, prioritize distinct target properties. The MARTINI water model is parametrized primarily to reproduce partitioning free energies between polar and apolar phases across a broad set of compounds, supporting transferability among lipids, proteins, and other biomolecules.^6,8^ By contrast, the SPICA water model is parametrized to reproduce experimental water density, surface and interfacial tension, together with solvation or transfer free energies of various solutes derived from AA-MD reference data, which are important for characterizing self-assembled surfactant and lipid structures.^13^ Recent comparative studies have also reported that the SPICA water model reproduces the experimental enthalpy of vaporization, isothermal compressibility, and self-diffusion coefficient more accurately than other widely used nonpolar CG water models.^23^

Although computationally efficient, uncharged water models with implicit dielectric screening cannot explicitly represent the local dielectric response of water. This limitation becomes important in processes involving strong local electric fields or charge-driven structural transitions, such as membrane electroporation.^29,30^ To address this issue, several CG water models incorporate explicit electrostatic interactions through permanent charge distributions or polarizable representations. These models typically distribute partial charges onto additional off-center sites that carry no van der Waals interaction, a design analogous to the charge-only M site of the TIP4P water model and to the dummy sites in cationic dummy-atom models of metal ions.^31,32^ Permanent-charge models embed fixed point charges within each CG water particle to generate an electric dipole and, in some cases, higher-order multipoles, thereby enabling an explicit response to the local electrostatic environment.^29,30,33–36^ The big multipole water (BMW) model uses three charged sites to reproduce both the dipole and quadrupole moment tensor of water clusters.^33,37^ The polar pSPICA water model employs a simpler two-site dipolar representation optimized within the SPICA parametrization framework.^30,38^ In contrast, the polarizable MARTINI water model contains a dipole that responds dynamically to the local electric field rather than remaining fixed.^29^ These charge-bearing models have been used to simulate membrane electroporation and ion transport across lipid bilayers, illustrating the importance of explicit electrostatic degrees of freedom in CG water representations.^29,30,39,40^

A separate and persistent challenge in CG water modeling is the occurrence of freezing artifacts. Systematic parameter-space scans have shown that the freezing tendency is an intrinsic limitation of many uncharged CG water models: the melting temperature (*T*_m_) generally increases with the level of coarse-graining and as the nonbonded potential becomes steeper or shorter-ranged.^41,42^ Both the SPICA and pSPICA water models use relatively strong van der Waals (vdW) interactions to reproduce the high surface tension of water and, as shown below, exhibit substantial freezing artifacts. To mitigate this problem, the standard MARTINI water model includes a small fraction of antifreeze particles with a slightly larger vdW radius. These particles disrupt local crystalline packing while preserving the parametrized bulk thermodynamic properties.^6^ The more recent Martini3 FF represents bulk water as a mixture of CG sites of three different sizes to address this persistent artifact.^8,43^ Maintaining a uniform mixture composition from the bulk to interfaces, however, remains challenging.^43^

By contrast, a comparable freezing artifact has not been reported for the BMW model, which uses a three-site dipole–quadrupole representation.^33,39,40^ This behavior may be related to the softer repulsive core of the Born-Mayer-Huggins potential used in that model.^33^ However, the BMW model was parametrized and validated at only one ambient temperature, which limits assessment of its performance over a wider temperature range.

The SPICA water model provides a useful basis for examining this issue because it reproduces several liquid-water properties, including density, surface and interfacial tensions, and solvation free energies, more accurately than several commonly used nonpolar CG water models.^13,23^ In addition, pSPICA introduces a two-site permanent dipole while retaining the accuracy of selected bulk-water properties.^30^ Together, these models provide a framework for examining how charge representation and nonbonded interaction parameters are associated with the freezing artifact. We considered three charge topologies—uncharged, two-site dipolar, and three-site multipolar—within the SPICA/pSPICA framework, and evaluated their melting behavior and freezing kinetics. Because each model was parametrized to reproduce the experimental density and surface tension, the charge topology and nonbonded parameters cannot be varied independently. The comparison therefore addresses how different charge representations, as a design degree of freedom under realistic parametrization constraints, affect the freezing behavior of CG water.

Permanent-charge CG water models commonly use a background dielectric constant, ε_bg_, to complement the explicit charge representation.^29,30,33^ However, the influence of ε_bg_ on the balance between electrostatic and vdW interactions has not been systematically examined in the context of freezing suppression. We therefore explored ε_bg_ as a design parameter and asked whether strengthening the explicit electrostatic contribution could permit a reduction in the LJ attraction while preserving selected target properties of liquid water. Our objective was to establish a parametrization strategy that mitigates the freezing artifact by rebalancing electrostatic and vdW interactions. Following this strategy, we developed pSPICA2, a new polar CG water model that has a substantially reduced *T*_m_ and retains the dielectric response and thermodynamic accuracy within the SPICA/pSPICA framework.

## Methods

### CG Water Models with Different Charge Topologies

In the SPICA FF, three water molecules are mapped onto a single uncharged bead, denoted W. The W beads interact through a pairwise LJ potential. To compensate for the absence of an explicit molecular dipole, a high background dielectric constant of ε_bg_ = 80 is applied uniformly, corresponding to the assumption that electrostatic interactions between hydrated polar solutes are strongly screened. SPICA therefore serves here as a nonpolar reference for assessing models with explicit charge distributions.^13,30^ The charge topologies of all models examined in this study are illustrated in Figure 1.

**Figure 1:**
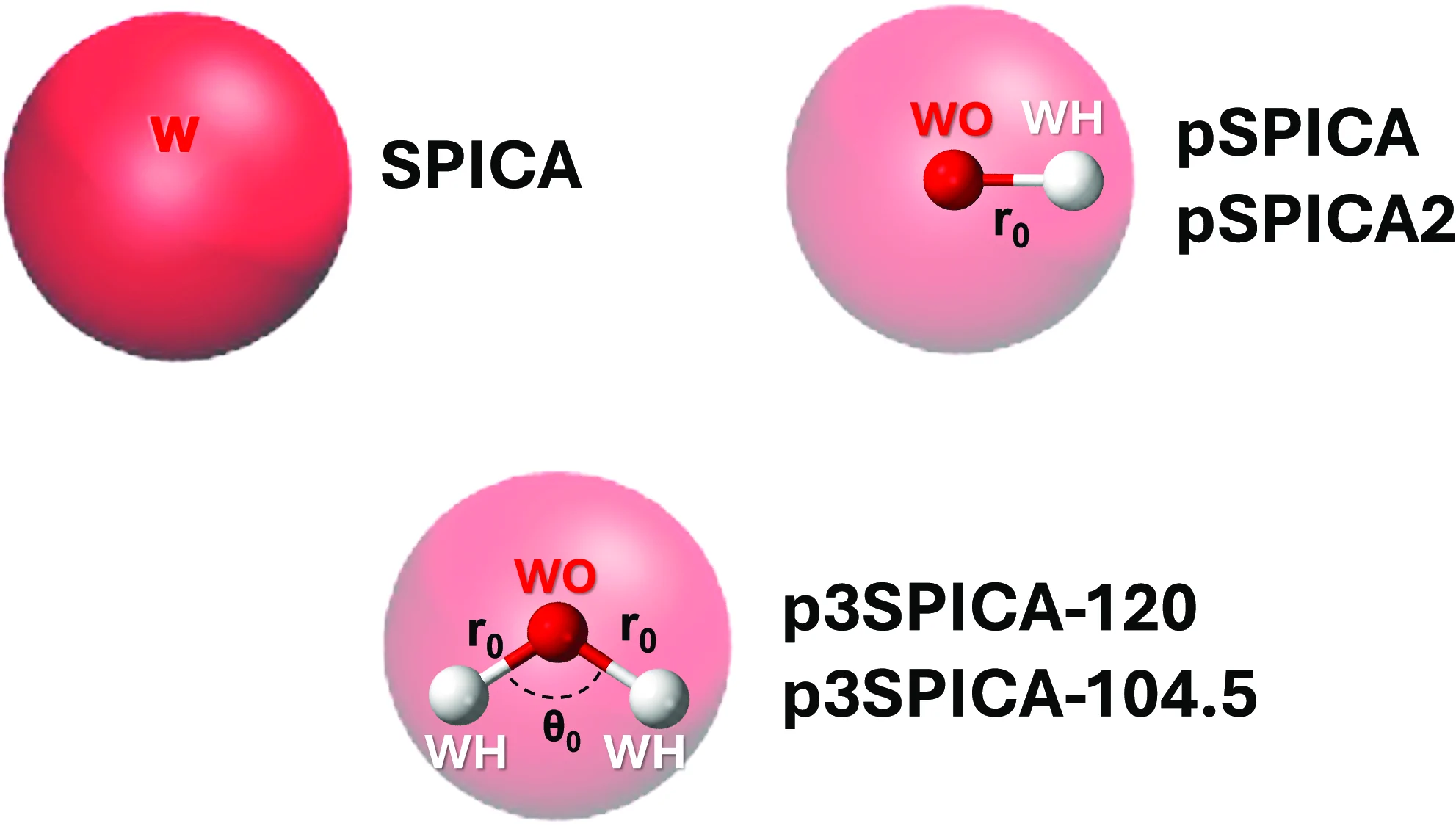
Schematic representations of the CG water models investigated in this work. SPICA employs a single uncharged bead (W). pSPICA and the newly proposed pSPICA2 utilize a linear two-site topology with a bond length (*r*_0_). p3SPICA-120 and p3SPICA-104.5 use three-site topologies with the same bond length (*r*_0_) and bond angles (θ_0_) of 120° and 104.5°, respectively. The opaque sphere in SPICA and the transparent spheres in the polar models represent the vdW radii of the W and central WO sites, respectively. The small red and white spheres in the polar models indicate the positions of the WO and WH charge sites, respectively. Force-field parameters for all models are listed in Table 1. The figure was generated using VMD.^44^

The pSPICA model introduces an explicit molecular dipole through a two-site representation while retaining the mapping ratio of three water molecules per CG particle. Each particle consists of a central WO bead and a satellite WH bead connected by a rigid bond of length *r*_0_, with partial charges of −0.836*e* and +0.836*e* assigned to WO and WH, respectively.^30^ The LJ interaction is only assigned to the WO–WO pairs, whereas electrostatic interactions are evaluated between all intermolecular site pairs; the intraparticle WO–WH pair is excluded. The bond length and partial charges were chosen to yield a dipole moment of 4.4 D, corresponding to the average dipole moment of a three-water-molecule cluster obtained from AA-MD simulations.^30^

With the introduction of the explicit dipole, ε_bg_ is reduced from 80 in SPICA to 3.2 in pSPICA. The LJ parameters were optimized to reproduce the experimental bulk density and air–water surface tension under ambient conditions, consistent with the SPICA parametrization strategy.^13,23,30^ As described below, we also developed pSPICA2, which uses the same two-site topology but rebalances the vdW and electrostatic interactions to mitigate the freezing artifact observed in pSPICA.

To examine charge representation beyond a linear dipole, we constructed three-site CG water models in which two WH beads are bonded symmetrically to a central WO bead with bond length *r*_0_ and an interbead angle θ_0_, forming a rigid V-shaped geometry analogous to that of an atomistic water molecule (Figure 1). This topology, which is also used in the BMW model,^24,33,37^ introduces a quadrupolar component that is absent from the two-site linear model while maintaining a net dipole moment of 4.4 D. We examined θ_0_ values of 120°, following the BMW model geometry, and 104.5°, corresponding to the experimental HOH bond angle of water. These models are denoted p3SPICA-120 and p3SPICA-104.5, respectively.

### Force Field Parameters and Simulation Details

In the SPICA and pSPICA FFs, the nonbonded vdW interactions between CG water beads are described by Lennard–Jones potentials of the LJ 12–4, LJ 12–5 or LJ 9–6 form. These potentials can be written in the generic LJ *m*–*n* form

1 $$U_{\mathrm{LJ}}\left(r\right)=A_{0}\epsilon_{\mathrm{LJ}}\left[{\left(\frac{\sigma_{\mathrm{LJ}}}{r}\right)}^{m}-{\left(\frac{\sigma_{\mathrm{LJ}}}{r}\right)}^{n}\right]$$

where σ_LJ_ denotes the vdW diameter parameter defined by *U*_LJ_(σ_LJ_) = 0, ϵ_LJ_ determines the strength of the vdW interaction, and $A_{0}=\frac{n}{m-n}{\left(\frac{m}{n}\right)}^{m/\left(m-n\right)}$ is the prefactor chosen to ensure the minimum of the LJ potential at σ_min_ = (*m*/*n*)^1/(*m*−*n*)^σ_LJ_ equals −ϵ_LJ_.

For SPICA water, the LJ 12–4 form (*m* = 12, *n* = 4) was selected for the W–W interaction because its steep repulsive term provided the best reproduction of liquid compressibility while simultaneously matching the experimental bulk density and surface tension under ambient conditions.^13,42^ Nonwater vdW interactions in the SPICA FF employ the LJ 9–6 form (*m* = 9, *n* = 6). In pSPICA, the interaction between two central WO sites is described by the LJ 12–5 form (*m* = 12, *n* = 5). The p3SPICA-120 and p3SPICA-104.5 models also use the LJ 12–5 form for the WO–WO interaction. Following the original pSPICA parametrization, ϵ_LJ_ and σ_LJ_ were calibrated to reproduce the experimental density and surface tension of water at 298 K.^30^ In all models, LJ interactions were truncated at *r*_cut_ = 15 Å. The complete LJ parameter sets are listed in Table 1.

**Table 1:** Force Field Parameters for the CG Water Models Investigated in This Work

Model	ε_bg_	LJ type *m*–*n*	ϵ_LJ_ (kcal/mol)	σ_LJ_ (Å)	*Q*_WO_ (e)	*Q*_WH_ (e)	*r*_0_ (Å)	θ_0_
SPICA	80	12–4	0.895	4.371	–	–	–	–
pSPICA	3.2	12–5	1.050	4.400	–0.836	0.836	1.100	–
p3SPICA-120	3.2	12–5	0.952	4.430	–1.248	0.624	1.474	120(°)
p3SPICA-104.5	3.2	12–5	0.994	4.410	–0.942	0.471	1.595	104.5(°)
pSPICA2	1.5	12–4	0.698	4.485	–0.541	0.541	1.700	–

Electrostatic interactions between all intermolecular charged-site pairs were calculated using the Coulomb potential

2 $$U_{\mathrm{coul}}\left(r\right)=\frac{Q_{i}Q_{j}}{4\pi \varepsilon_{0}\varepsilon_{\mathrm{bg}}r}$$

where *Q*_*i*_ and *Q*_*j*_ are the partial charges on sites *i* and *j*, ε_0_ is the permittivity of vacuum, and ε_bg_ is the background dielectric constant. In the nonpolar SPICA model, ε_bg_ = 80 is used to compensate for the absence of explicit charge sites and to represent strong electrostatic screening in a hydrated environment. In pSPICA, ε_bg_ = 3.2 is used to represent residual screening.^30^ The p3SPICA-120 and p3SPICA-104.5 models use the same value, ε_bg_ = 3.2, so that comparisons among the polar models are not additionally complicated by differences in the background dielectric constant. Short-range electrostatic interactions were calculated within the LJ cutoff of 15 Å, while long-range electrostatic interactions were evaluated using the particle–particle particle–mesh (PPPM) method.^45^ All intraparticle electrostatic interactions were excluded from the electrostatic calculation.

The purpose of incorporating a fixed dipole into each pSPICA water particle is to provide an explicit response to the local electrostatic environment. The original pSPICA parametrization yielded a relative dielectric permittivity, ε_r_, of 80.7, calculated from equilibrium fluctuations of the total dipole moment using the Kirkwood–Froehlich relation^46^

3 $$\varepsilon_{r}=1+\frac{\langle M^{2}\rangle -\langle M\rangle^{2}}{3\varepsilon_{0}Vk_{B}T}$$

where *M* denotes the total dipole moment of the system, *k*_B_ is the Boltzmann constant, and *V* and *T* are the system volume and temperature, respectively.

The BMW model instead used a PMF-based approach and reported an effective screening constant of ε_eff_ = 74.^33^ The use of a PMF-based measure was motivated by the explicit background dielectric constant, ε_bg_ = 1.3, in the BMW model. Because pSPICA similarly combines explicit dipoles with ε_bg_ = 3.2, the physical interpretation of the fluctuation-derived ε_r_ should be verified rather than assumed. We therefore treat ε_r_ from eq 3 as an operational measure of the bulk dielectric response under the chosen Hamiltonian and compare it with an independently estimated ε_eff_.

A direct PMF fit is less straightforward for SPICA water because the steep short-range repulsion produces pronounced solvent layering and corresponding oscillations in the PMF. To reduce the influence of these spatial oscillations, we measured the mean force between two test particles held at a fixed separation *r* while varying the magnitude *Q* of their charges. At a sufficiently large fixed separation, the mean force was fitted to

4 $$\left\langle F\left(Q\right)\right\rangle =\frac{Q^{2}}{4\pi \varepsilon_{0}\varepsilon_{\mathrm{eff}}r^{2}}+C$$

where ⟨⟩ denotes the ensemble average, and the constant *C* represents the charge-independent contribution from solvent structure at the selected separation. The separation was set to 32 Å, close to half the simulation-box length. This procedure assumes that the solvent-structure contribution is approximately independent of *Q* over the fitted charge range. The resulting ε_eff_ therefore represents the effective screening constant under the employed periodic boundary conditions. In addition, because the surrounding solvent may still reorganize in response to large test charges, ε_eff_ was used as an independent, qualitative cross-check of the fluctuation-derived ε_r_ rather than as a higher-precision dielectric estimate.

When the background dielectric constant ε_bg_ = 1, the mean-force-derived ε_eff_ should equal the fluctuation-derived ε_r_. To test such consistency, we performed an additional simulation in which all pSPICA partial charges were scaled by $1/\sqrt{\varepsilon_{\mathrm{bg}}}$ with ε_bg_ = 3.2 while the background dielectric constant was reset to 1.0. The electrostatic force is then formally unchanged because

5 $$F_{\mathrm{coul}}\left(r\right)=\frac{Q_{i}Q_{j}}{4\pi \varepsilon_{0}\varepsilon_{\mathrm{bg}}r^{2}}=\frac{Q_{i}^{\prime }Q_{j}^{\prime }}{4\pi \varepsilon_{0}\varepsilon_{\mathrm{bg}}^{\prime }r^{2}}$$

where $Q^{\prime }=Q/\sqrt{\varepsilon_{\mathrm{bg}}}$ is the scaled charge and ε_bg_^′^ = 1 for the rescaled simulation. For this rescaled system, the dielectric permittivity ε_r_^′^ obtained from dipole fluctuations can be compared directly with the effective screening constant ε_eff_^′^ obtained from the force-fitting method. This comparison tests whether the two operational measures are mutually consistent. The comparison was then extended to the pSPICA system with ε_bg_ = 3.2 to cross-check the resulting ε_eff_ and ε_r_ values.

The intramolecular geometry of each polar CG water model was maintained using the SHAKE algorithm.^47^ For the two-site pSPICA model, the WO–WH bond length *r*_0_ was constrained. For p3SPICA-120 and p3SPICA-104.5, the two WO–WH bond lengths and the WH–WO–WH angle θ_0_ were constrained simultaneously to maintain a rigid V-shaped geometry. As described below, the partial charges and bond length were adjusted jointly to reproduce a target static relative dielectric permittivity of approximately 80 while maintaining a net dipole moment of 4.4 D, as in pSPICA.^30^ The force-field parameters of pSPICA2 were determined using the reparameterization procedure described in the Results and Discussion section. The resulting parameter sets for all models are summarized in Table 1.

All CG-MD simulations were performed with LAMMPS.^48,49^ Bulk-water systems contained 4800 CG water particles in cubic simulation cells with periodic boundary conditions in all three dimensions. Temperature was controlled using a Nosé–Hoover thermostat with a relaxation time of 1 ps, and pressure was controlled using a Parrinello–Rahman barostat with a relaxation time of 10 ps.^50–52^ The equations of motion were integrated using the velocity-Verlet algorithm with a time step of 10 fs. Bulk-water simulations were conducted in the NPT ensemble at 1 atm over the temperature range of 290–363 K to obtain the density thermogram ρ­(*T*).

Surface tension was calculated using a water-slab geometry. Starting from an equilibrated bulk configuration, the simulation box was extended to 150 Å along the *z* direction, creating two vacuum–water interfaces normal to the *z* axis. These simulations were conducted in the NVT ensemble. The surface tension, γ, was calculated from the diagonal components of the pressure tensor as

6 $$\gamma =\frac{L_{z}}{2}\left\{\langle P_{\mathrm{zz}}\rangle -\frac{\left\langle P_{\mathrm{xx}}\right\rangle +\left\langle P_{\mathrm{yy}}\right\rangle }{2}\right\}$$

where *L*_*z*_ is the box length in the *z* direction and *P*_*xx*_, *P*_*yy*_, and *P*_*zz*_ are the diagonal components of the pressure tensor. The factor of 1/2 accounts for the two equivalent interfaces present in the slab system.

### Characterization of the Operational Melting Temperature and Nucleation Dynamics

The melting behavior of each CG water model was characterized using a direct solid–liquid coexistence protocol.^53^ Two simulation cells containing 1200 CG water particles each were prepared: one was crystallized at low temperature to obtain a fully solid configuration, and the other contained liquid water equilibrated at 298 K and 1 atm. The cells were joined along the *z* direction to produce a 2400-particle system with the solid–liquid interface normal to *z*. Before the coexistence simulation, the combined system was energy-minimized and equilibrated for 10 ps to relax the interface. Production simulations were then performed in the NPT ensemble at 1 atm using an anisotropic barostat that allowed the three box dimensions to fluctuate independently, thereby permitting the solid phase to adopt its equilibrium lattice dimensions and minimizing artificial strain on the solid that could bias the measured *T*_m_.

Starting from the combined solid–liquid configuration, independent equilibrium simulations were performed at a series of temperatures in 1 K increments. In this work, *T*_m_ was then defined as the lowest tested temperature at which the solid phase melted completely within the simulation time window. Because this criterion depends on the system size, the simulation time window, and the initial interface, the resulting *T*_m_ should be interpreted as the operational *T*_m_ rather than the exact equilibrium melting temperature. All models were nevertheless evaluated with an identical protocol, so the relative *T*_m_ values remain a valid basis for comparing freezing tendency.

To characterize the propensity of each water model to freeze, nucleation simulations were performed at 240 K, a temperature sufficiently below the operational *T*_m_ values of the original models to produce spontaneous crystallization within accessible simulation times. Each simulation was initialized from a liquid configuration of 1200 CG water particles pre-equilibrated at 298 K and 1 atm. Fifty starting configurations were extracted from a 10 ns pre-equilibration trajectory. Each configuration was quenched to 240 K and simulated at 1 atm using an anisotropic barostat, with the thermostat and barostat settings described above.

The growth of crystalline nuclei was monitored using a bond-orientational order parameter to classify CG water particles as solid-like or liquid-like.^54,55^ For each particle *i*, the complex order-parameter vector *q̅*_6*m*_(*i*) was calculated using the freud package,^56,57^ as

7 $${\mathrm{q̅}}_{6m}\left(i\right)=\frac{1}{N_{b}\left(i\right)}\sum_{j\in N\left(i\right)}Y_{6m}\left({\mathrm{r̂}}_{\mathrm{ij}}\right)$$

where *Y*_6*m*_ denotes a spherical harmonic of degree *l* = 6 and *r̂*_*ij*_ is the unit vector from particle *i* to particle *j*. The sum extends over the *N*_*b*_(*i*) neighbors within 6.0 Å, corresponding to the first minimum of the radial distribution function between CG water particles. Particles *i* and *j* were defined as having a crystalline connection when the normalized dot product of their order-parameter vectors satisfies

8 $$\frac{\sum_{m=-6}^{6}{\mathrm{q̅}}_{6m}\left(i\right){\mathrm{q̅}}_{6m}^{*}\left(j\right)}{\left|{\mathrm{q̅}}_{6}\left(i\right)\right|\left|{\mathrm{q̅}}_{6}\left(j\right)\right|}\geq 0.7$$

A particle was then classified as solid-like when it formed at least seven crystalline connections with its neighbors.^54,55^ The largest contiguous cluster of solid-like particles in each frame was identified as the nucleus, and its size *n* was used as the nucleation order parameter.

From the 50 independent freezing trajectories, we calculated the mean first-passage time (MFPT), τ­(*n*), defined as the average time required for the largest crystalline nucleus to reach size *n* for the first time.^58,59^ To obtain comparative nucleation parameters, τ­(*n*) was fitted to the sigmoidal function with the crystal-growth correction proposed by Yuhara et al.^60^

9 $$\tau \left(n\right)=\frac{\tau_{n}}{2}\left[1+\mathrm{erf}\left(\left(n-n^{*}\right)Z\sqrt{\pi }\right)\right]+\frac{1}{2R_{G}}\left(n-n^{*}\right)\left[1+\mathrm{erf}\left(C\left(n-n^{*}\right)\right)\right]$$

where τ_*n*_ is the critical nucleation time, n* is the fitted critical nucleus size, *Z* is the Zeldovich factor, *R*_G_ is the postcritical crystal-growth rate, and *C* = 5 is a smoothing constant that activates the growth-correction term in the postnucleation regime. The nucleation free energy barrier was then estimated from the fitted parameters as ΔG*/*k*_B_ *T* = 3π­(n*)^2^ *Z* ^2^, following the classical-nucleation-theory-based treatment of Yuhara et al.^60^ All models were simulated under an identical protocol. The fitted ΔG* and *R*_G_ therefore provide a consistent basis for comparing freezing behavior among the models, although their absolute values should not be interpreted as macroscopic nucleation parameters.

## Results and Discussion

### Comparison of CG Water Models with Different Charge Topologies

We first compared the bulk thermodynamic properties and effective dielectric screening of SPICA, pSPICA, p3SPICA-120, and p3SPICA-104.5. The parameters listed in Table 1 were selected to reproduce the experimental density and surface tension at 298 K. Figure 2 shows the temperature dependence of the bulk density, ρ­(*T*), and air–water surface tension, γ­(*T*), for these models, together with the corresponding experimental values over 290–363 K.

**Figure 2:**
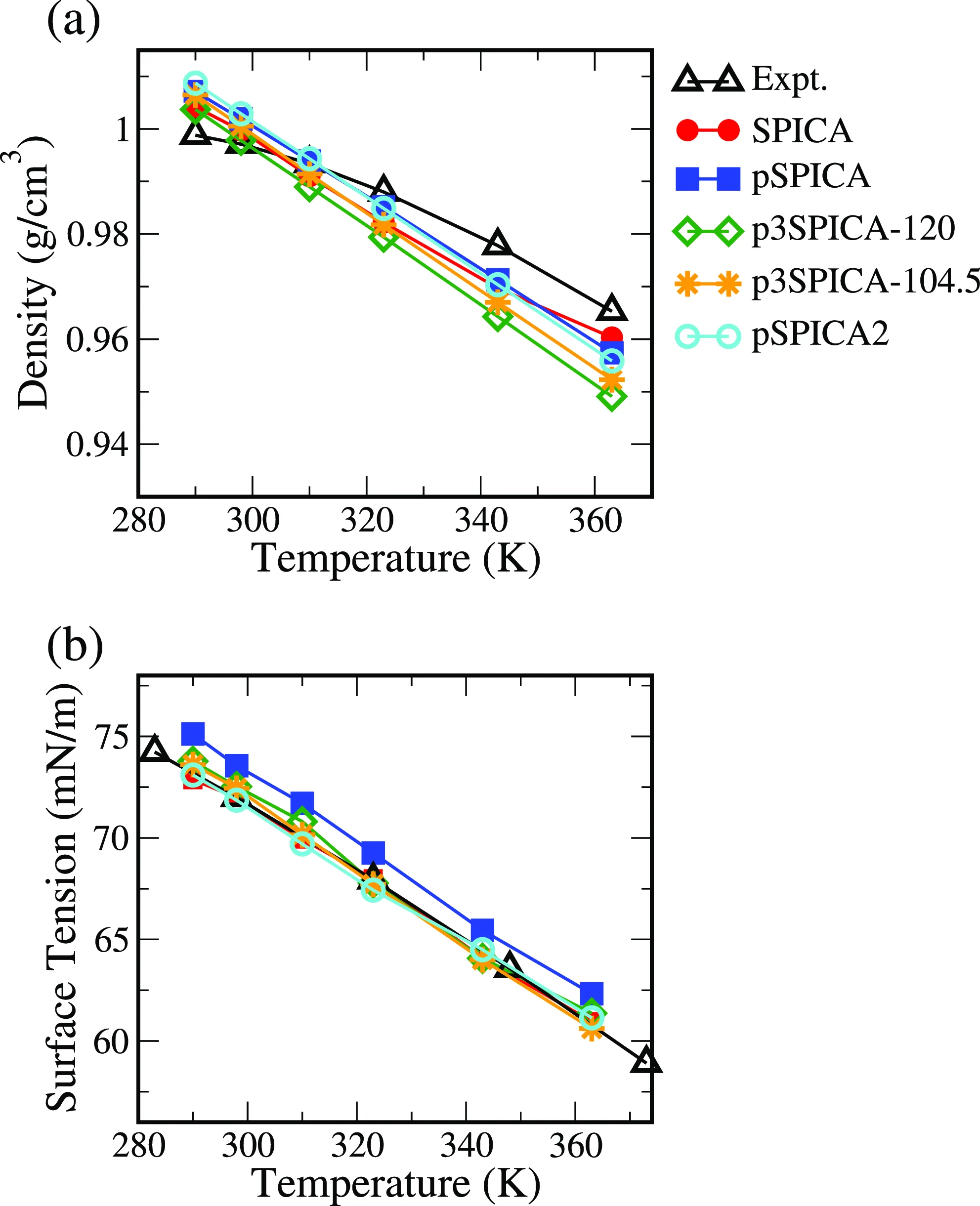
Temperature dependence of (a) density and (b) surface tension of the CG water models SPICA, pSPICA, p3SPICA-120, and p3SPICA-104.5. The newly parametrized two-site polar CG water model, pSPICA2, is also included for comparison. Experimental values (Expt.) are shown for ref 61.

All four models reproduce the monotonic decrease in water density with increasing temperature, in qualitative agreement with experiment. Near 298 K, the calculated densities are within approximately 1% of the experimental value (∼0.997 g/cm^3^), indicating that all parameter sets reproduce the ambient density accurately. The ρ­(*T*) profiles are also broadly similar among the models. All models show the same principal deviation from experiment: a systematic positive offset at lower temperatures and an underestimation at higher temperatures, with the two three-site models showing the greater high-temperature deviations. Because all models were parametrized with different nonbonded parameters to reproduce the density at 298 K, the observed deviations emerge outside the target state point. Such deviation may arise from the charge topology and its associated orientational correlations, the fitted nonbonded parameters, or their combined effects.

The surface-tension results show a similarly modest dependence on charge representation. The experimental slope dγ/d*T* is preserved reasonably well across all charge representations over the examined temperature range, in contrast to the model-dependent divergence seen in ρ­(*T*) at elevated temperatures. The main difference is an upward offset of approximately 2–3 mN/m for pSPICA relative to the experiment and to the other models. This offset is more plausibly attributed to the specific parametrization than to the two-site topology itself.

We next evaluated the dielectric response of pSPICA by comparing the effective screening constant, ε_eff_, obtained from the mean-force method (eq 4) with the relative dielectric permittivity, ε_r_, obtained from dipole fluctuations (eq 3). Calculations were performed both for the standard pSPICA model with ε_bg_ = 3.2 and for the charge-scaled system with ε_bg_^′^ = 1.0 as shown in Figure 3. For the charge-scaled system, the fitting of the mean force yielded ε_eff_^′^ = 27.79, in good agreement with ε_r_^′^ = 26.57 from the fluctuation method (eq 3). For standard pSPICA, the corresponding values were ε_eff_ = 85.27 and ε_r_ = 80.7. Within the statistical precision of force measurements and the limitations of the finite periodic test system, this agreement supports the use of the fluctuation-derived ε_r_ as an operational measure of dielectric screening in pSPICA.

**Figure 3:**
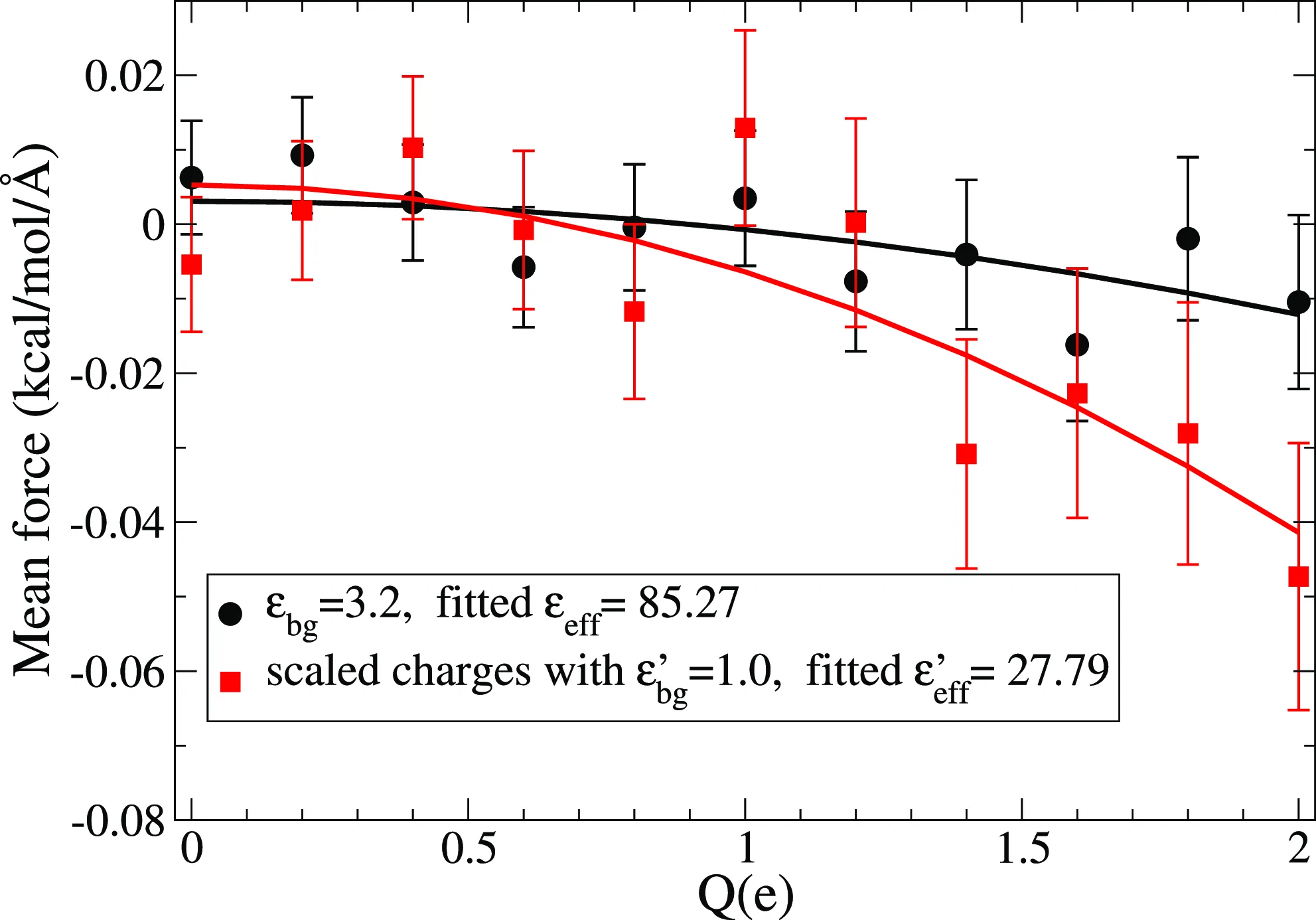
Mean force between two test charges as a function of the charge magnitude (*Q*) at a fixed separation of (*r* = 32) Å for pSPICA with the default (ε_bg_ = 3.2) (black circles) and for the scaled-charges pSPICA system with (ε_bg_^′^ = 1.0) (red squares). Error bars represent standard errors of the mean force estimated by bootstrapping. Solid lines represent fits to eq 4 used to determine the effective screening constants (ε_eff_).

Both ratios ε_eff_/ε_eff_^′^ ≈ 3.07 and ε_r_/ε_r_^′^ ≈ 3.04 are close to ε_bg_ = 3.2. This numerical agreement is consistent with the expected scaling of the direct Coulomb term for the two specifically compared Hamiltonians and with the independent analysis of Chiacchiera et al.^62^ However, whether this linear scaling extends beyond the two parameter sets examined here requires further validation, since changing ε_bg_ also alters the solvent structure and collective orientational correlations.

The mean-force data in Figure 3 remained substantially scattered even after 50 ns of sampling for each test-charge magnitude, demonstrating the slow convergence of force-based estimates. These fluctuations limit the precision of the fitted ε_eff_. We therefore used the mean-force analysis primarily to cross-check the fluctuation method and used eq 3 for subsequent dielectric-permittivity calculations because it converged more readily under the present conditions.

In the two-site pSPICA model, *Q*_WO_ = −0.836*e*, *Q*_WH_ = +0.836*e*, and *r*_0_ = 1.1 Å give a fixed dipole moment of 4.4 D, corresponding to the average dipole moment of a three-water-molecule cluster in the AA-MD reference simulations.^30^ The p3SPICA-120 and p3SPICA-104.5 models were likewise constrained to a net dipole moment of 4.4 D. At fixed net dipole moment, the calculated dielectric permittivity nevertheless depends on *r*_0_. As shown in Figure 4, increasing *r*_0_ from 1.0 to 1.5 Å decreased ε_r_ from 89 to 61, whereas the density and surface tension changed only slightly over the same range.

**Figure 4:**
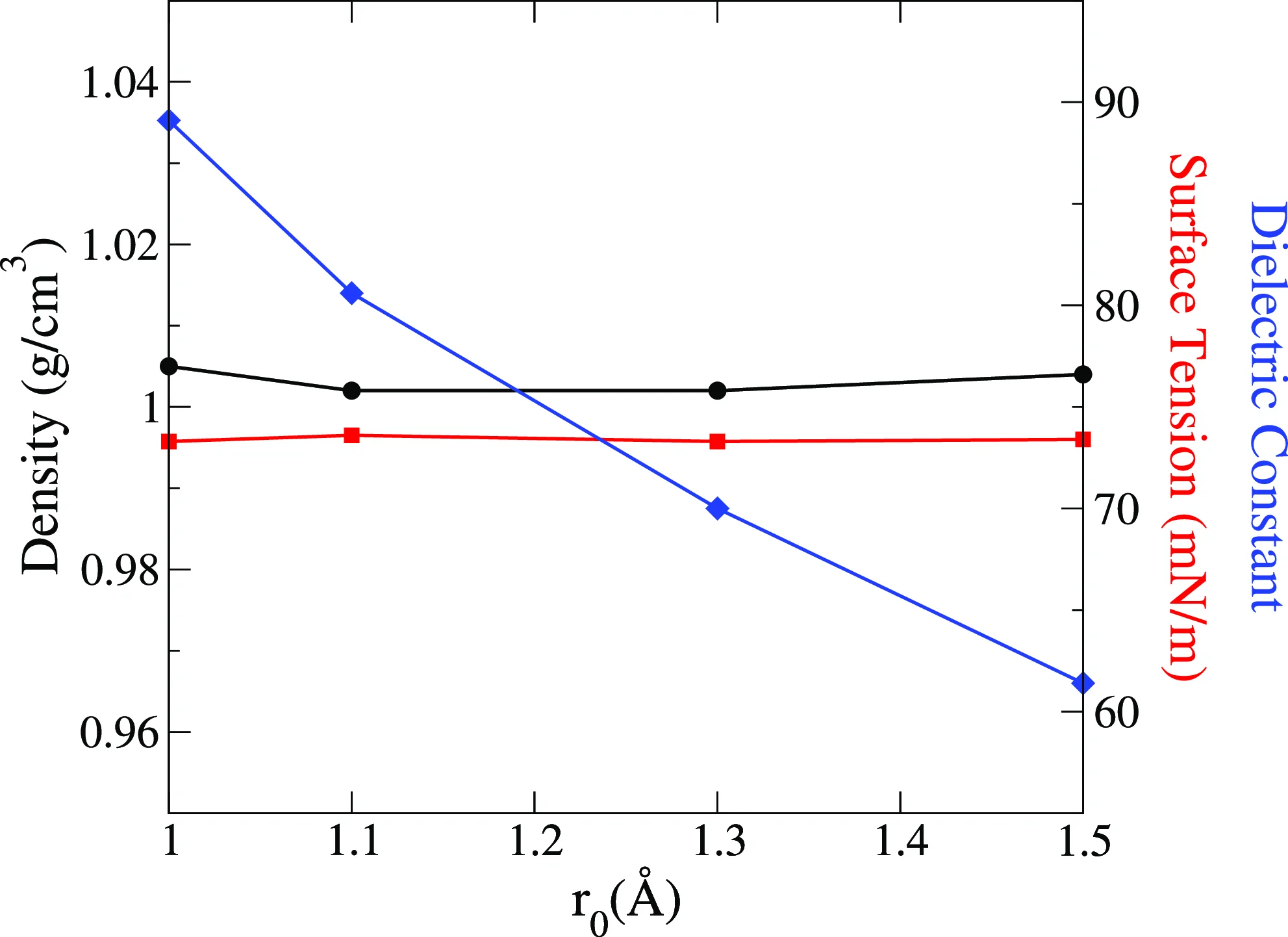
Bulk density (black circles), surface tension (red squares), and relative dielectric permittivity (blue diamonds) of the pSPICA model as functions of the bond length (*r*_0_), varied from 1.0 Å to 1.5 Å. The magnitudes of the partial charges (*Q*) were adjusted inversely with (*r*_0_) to maintain a fixed dipole moment of 4.4 D.

This result shows that *r*_0_ provides a useful parameter for tuning collective dipole fluctuations with comparatively little effect on the fitted mechanical properties over the range examined. Varying *r*_0_ at a fixed net dipole moment alters the spatial charge distribution, molecular geometry, and orientational correlations. Any of these may contribute to the variation in system dipole fluctuation and hence the static dielectric permittivity.

Table 2 summarizes the bulk-water properties of the four models at 298 K. The calculated density, surface tension, and relative dielectric permittivity agree closely with the corresponding experimental values for all parameter sets, with deviations within approximately 1% for ρ and 3% for γ and ε_r_. Thus, the selected two- and three-site parametrizations reproduce the targeted ambient liquid properties to comparable accuracy. The agreement at 298 K therefore shows that the nonbonded parameters can be refitted for different charge representations to reproduce the target liquid properties.

**Table 2:** Bulk-Water Properties and Ice-Nucleation Parameters of the CG Water Models^a^

	ρ (g/cm^3^)	γ (mN/m)	ε_r_	*T*_m_ (K)	ΔG* (kcal/mol)	n*	τ_n_ (ps)	*R*_G_ (1/ps)
Expt.^61^	0.997	72.0	78.4	273				
SPICA	1.000	72.0		339	1.70	23.3	249.1	3.56
pSPICA	1.002	73.6	80.7	384	0.96	15.9	66.8	3.16
p3SPICA-120	0.998	72.5	80.0	360	1.50	17.2	150.1	1.93
p3SPICA-104.5	1.001	72.4	78.4	378	1.16	17.2	106.6	2.50
pSPICA2	1.003	71.9	79.1	302	-	-	-	-

a Bulk density (ρ), surface tension (γ), and relative dielectric permittivity (ε_r_) were evaluated at 298 K, whereas the melting temperature (*T*_m_) was determined using the solid-liquid coexistence protocol. Experimental values are included for comparison. Ice-nucleation parameters, including the nucleation free-energy barrier (ΔG*), critical nucleus size (n*), critical nucleation time (*τ*_n_), and crystal growth rate (*R*_G_), were obtained from simulations at 240 K. For pSPICA2, freezing was observed in only one of 50 independent simulations at 240 K using the same protocol as for the other models. Consequently, the nucleation parameters for pSPICA2 were not evaluated.

### Effects of Charge Representation on the Freezing Artifact of CG Water

Despite their similar ambient liquid properties, the four models exhibit markedly different operational *T*_m_ as listed in Table 2. All values exceed the experimental melting temperature of 273 K, confirming a pronounced freezing tendency within the SPICA-family models under the present coexistence protocol. SPICA has the lowest operational *T*_m_, 339 K, whereas pSPICA has the highest, 384 K. The p3SPICA-120 and p3SPICA-104.5 values are intermediate at 360 and 378 K, respectively. The polar models therefore have higher operational melting temperatures than nonpolar SPICA among the parameter sets examined.

Because the models differ not only in charge topology but also in LJ functional form and fitted LJ parameters under the parametrization constraints of density and surface tension, the higher *T*_m_ of the polar models cannot be attributed uniquely to the charge topology. Redistributing the charges over a V-shaped geometry lowers *T*_m_ relative to two-site pSPICA, substantially for p3SPICA-120 and only modestly for p3SPICA-104.5. Yet *T*_m_ of both three-site models remain well above that of nonpolar SPICA. The key factor governing *T*_m_ is examined in the following section.

To further compare freezing behavior, we calculated the mean first-passage time (MFPT) τ as a function of the largest crystalline nucleus size at 240 K from 50 independent trajectories. Figure 5 shows sigmoidal MFPT profiles for all four models, with an initial increase followed by a plateau after the nucleus passes the fitted critical size n*. The magnitude and shape of the curves differ substantially, indicating distinct nucleation and growth dynamics under the common quench protocol.

**Figure 5:**
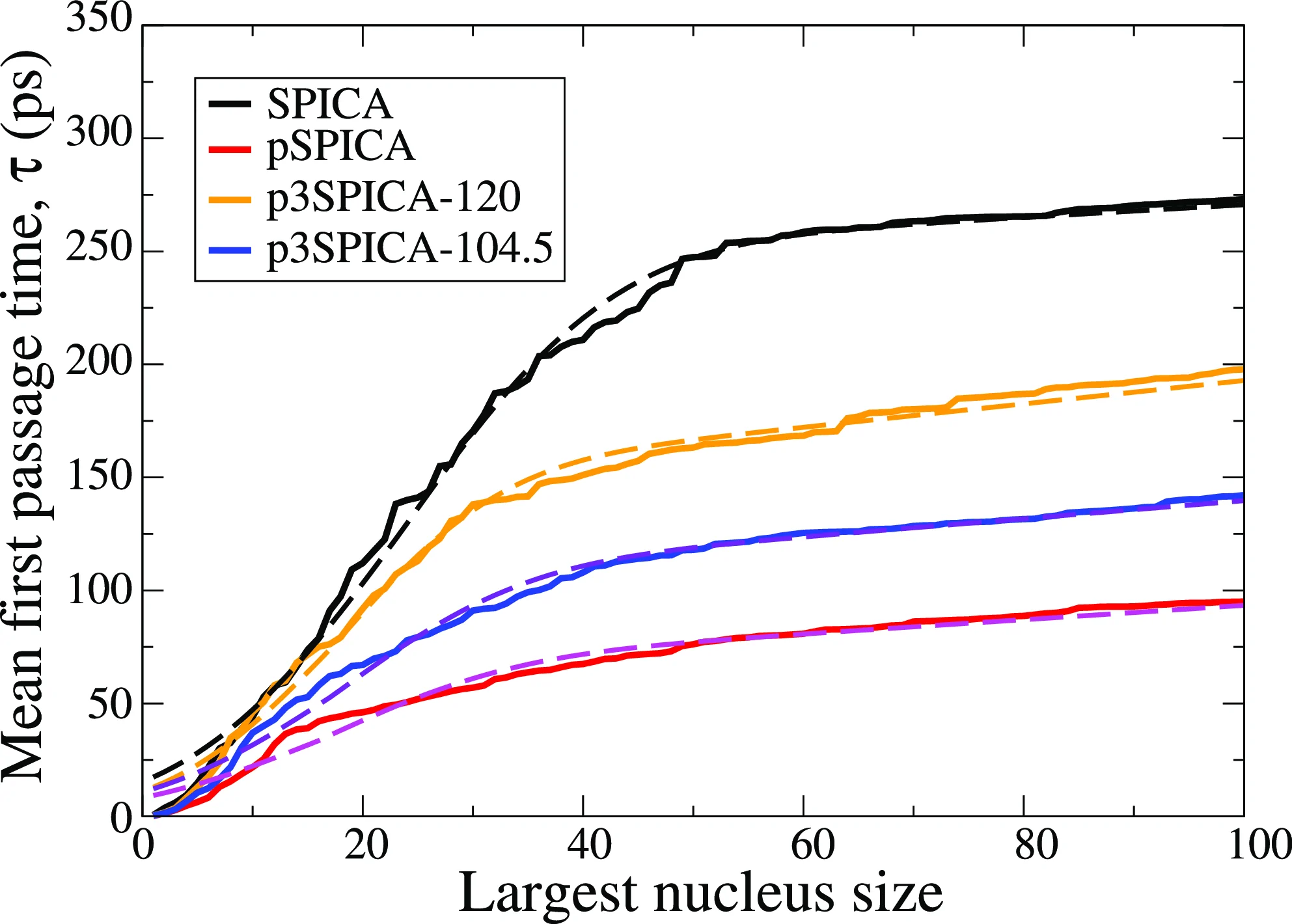
Mean first-passage time (MFPT), τ, as a function of the size of the largest nucleus, *n*, for SPICA, pSPICA, p3SPICA-120, and p3SPICA-104.5 at 240 K. The size of the largest nucleus is defined as the number of W or WO beads comprising the largest crystalline cluster, as described in the Methods section. Solid lines represent the simulation data, while dashed lines show fits of the MFPT data to eq 9. The nucleation free energy barrier (ΔG*), critical nucleus size (n*), critical nucleation time (τ_n_), and crystal growth rate (*R*_G_) extracted from the fits are summarized in Table 2.

The MFPT-derived parameters in Table 2 broadly follow the ordering of the operational melting temperatures. pSPICA has the smallest fitted ΔG*, n*, and τ_n_, whereas SPICA has the largest values. At the common quench temperature of 240 K, a model with a higher *T*_m_ experiences greater supercooling relative to its own melting point and therefore generally has a larger thermodynamic driving force for crystallization. The difference provides a straightforward explanation for at least part of the ordering in ΔG*, n*, and τ_n_. Charge representation and the fitted nonbonded interactions may also influence the solid–liquid interfacial free energy and local ordering pathways. Separating these contributions would require calculations beyond the present comparative MFPT analysis.

The postcritical growth rate *R*_G_ shows a different ordering: SPICA > pSPICA > p3SPICA-104.5 > p3SPICA-120, with SPICA giving *R*_G_ = 3.56 ps^–1^ and the three-site models giving the lowest values. This trend is consistent with an orientational contribution to particle attachment. The isotropic nonpolar SPICA particles can attach to a growing crystalline cluster without first satisfying an explicit charge orientation. A fixed linear dipole introduces an orientational constraint, whereas a rigid V-shaped charge distribution introduces additional geometric specificity. These factors may slow postcritical growth in the polar models. The lower *R*_G_ of p3SPICA-120 relative to p3SPICA-104.5 is also consistent with a greater orientational or packing penalty for the more open geometry, although the present data do not resolve this finer distinction.

Taken together, the coexistence and MFPT results show that models with similar ambient density, surface tension, and dielectric response can nevertheless have very different freezing behavior. The ordering of *T*_m_ does not follow the charge topology, whereas *R*_G_ decreases systematically from the isotropic model to the linear dipole and then to the two V-shaped models. Charge topology therefore appears to act mainly on the kinetics of postcritical growth rather than on *T*_m_. Changing the charge topology alone did not eliminate the freezing artifact in any of these models. This motivated us to examine the balance between the vdW and electrostatic interactions directly.

### Mitigation of the Freezing Artifact by Rebalancing vdW and Electrostatic Interactions

The interaction between two polar CG water particles contains both vdW and electrostatic contributions, represented here by LJ and Coulomb potentials, respectively. The parameters ϵ_LJ_ and σ_LJ_ control the magnitude and length scale of the vdW interaction, whereas ε_bg_ scales the direct electrostatic interaction. These contributions must be adjusted jointly when density and surface tension are used as parametrization targets because both properties depend on the total nonbonded interaction.

Under the parametrization constraints of reproducing the experimental density and surface tension, the LJ parameters vary among models with different charge topologies, particularly in ϵ_LJ_. The ϵ_LJ_ values of the four models span 0.895–1.050 kcal/mol, and their *T*_m_ values increase monotonically over this range as shown in Figure 6(a), despite the differences in charge topology and LJ functional form. Across these independently parametrized models, the ordering of *T*_m_ follows ϵ_LJ_ rather than the charge topology.

**Figure 6:**
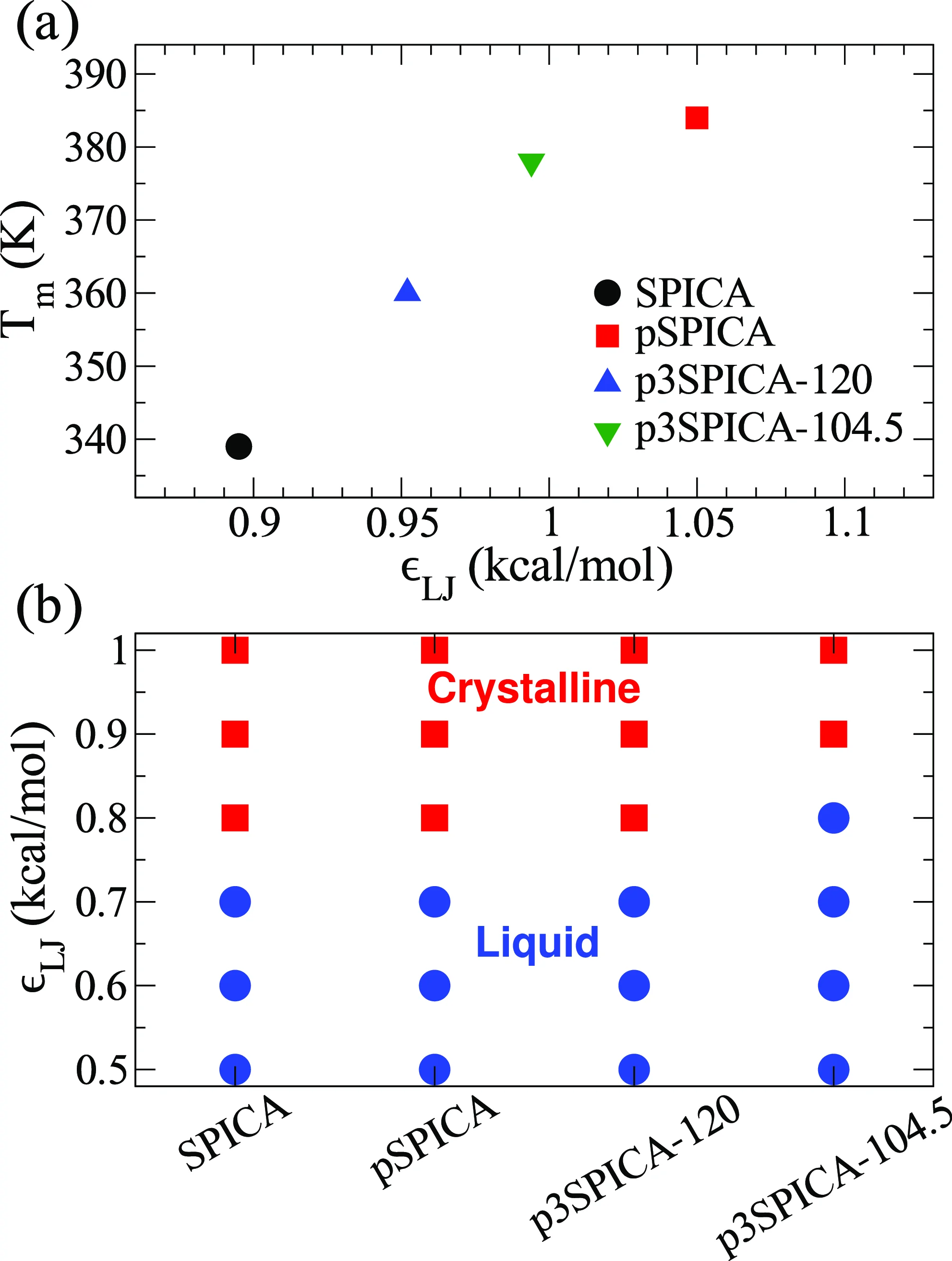
(a) Operational melting temperature *T*_m_ of the four CG water models as a function of their parametrized ϵ_LJ_ values. (b) Phase behavior at 273 K as a function of ϵ_LJ_ with 0.1 kcal/mol increments, and with all other force-field parameters held fixed. Red squares and blue circles indicate crystalline and liquid states, respectively.

To examine the role of the LJ attraction more directly, we performed solid–liquid coexistence simulations at 273 K for SPICA, pSPICA, p3SPICA-120, and p3SPICA-104.5 while varying ϵ_LJ_ and holding the other force-field parameters fixed. As shown in Figure 6(b), each model changed from liquid-like to crystalline behavior over a relatively narrow ϵ_LJ_ interval of approximately 0.7–0.9 kcal/mol, with p3SPICA-104.5 showing a slightly higher transition range. Under this specific protocol, ϵ_LJ_ = 0.7 kcal/mol therefore provides a practical lower-bound reference below which the liquid state was stable for all four models. This threshold was determined under the SPICA-family parametrization protocol, and its generality across other CG water models remains to be examined.

Nevertheless, the similar transition ranges across models with different charge representations indicate that the freezing behavior is highly sensitive to the LJ interaction strength. The parametrized ϵ_LJ_ values of the four original models lie above the liquid-stability reference identified in Figure 6(b), consistent with their high operational melting temperatures. Lowering ϵ_LJ_ toward this range should therefore mitigate freezing. To preserve the target density and surface tension while weakening the LJ attraction, the electrostatic contribution must be strengthened. With the dipole moment fixed at the AA-MD reference value of 4.4 D,^30^ we treat ε_bg_ as the parametrization variable that controls the weight of the electrostatic interaction within the total nonbonded interaction. Reducing ε_bg_ at a fixed dipole moment increases this electrostatic contribution and thereby permits a weaker LJ attraction, that is, a smaller ϵ_LJ_.

On this basis, we developed the parametrization framework shown in Figure 7 for introducing a permanent dipole into a nonpolar CG water model while reducing its freezing tendency. Following the framework, we began with nonpolar SPICA water and identified the threshold ϵ_LJ_ value for freezing, ϵ_thres_, which is 0.7 kcal/mol for the SPICA family as shown earlier. We then introduced the fixed 4.4 D dipole used in the original pSPICA parametrization.^30^ Various combinations of *ε*_bg_, ϵ_LJ_, and σ_LJ_ were then examined to quantify their relationships with the density and surface tension at 298 K. Over the parameter range examined, linear regressions were found to describe these local relationships adequately, as discussed in the Supporting Information. The fitted relations were used to identify parameter combinations that reproduced target values of ρ = 1 g/cm^3^ and γ = 72 mN/m at 298 K.

**Figure 7:**
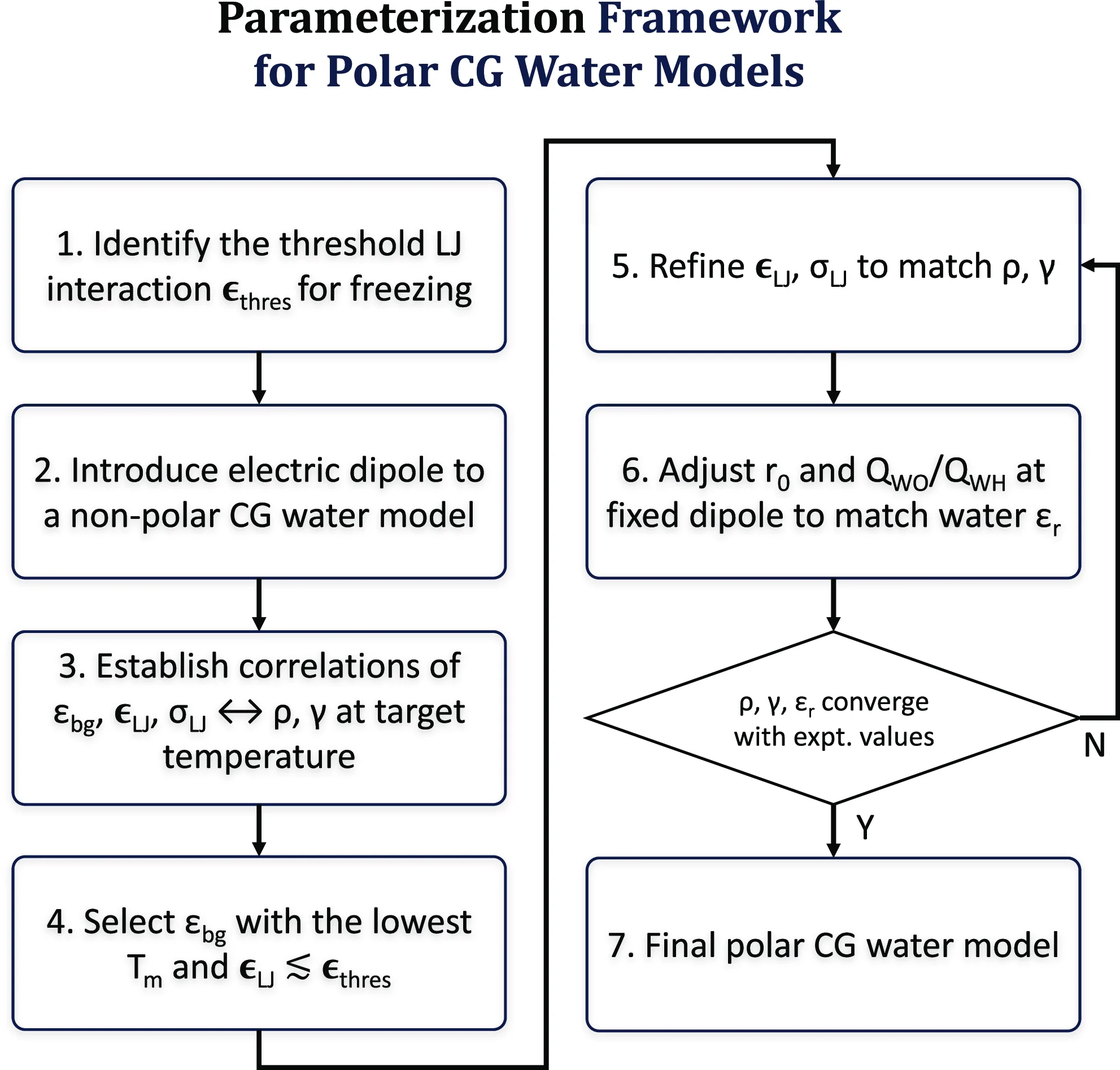
Proposed parametrization framework for introducing polarity into a nonpolar CG water model while mitigating its freezing tendency.

With the LJ 12–5 functional form used in pSPICA, however, we were unable to reproduce both target properties after reducing ϵ_LJ_ close to ϵ_thres_ = 0.7 kcal/mol. We therefore adopted the LJ 12–4 form used in nonpolar SPICA. Its longer-ranged attractive component provided sufficient flexibility to reproduce the density and surface tension with a weaker well depth, as detailed in the Supporting Information.

Reducing ε_bg_ strengthens the explicit electrostatic interaction and thus permits a smaller ϵ_LJ_ while retaining the target liquid properties. To assess the associated freezing behavior, we performed coexistence simulations for parameter sets spanning a range of ε_bg_ values, with ϵ_LJ_ and σ_LJ_ predicted from the fitted property relationships. Figure 8 shows a nonmonotonic dependence of the operational *T*_m_ on ε_bg_, with a minimum near ε_bg_ = 1.5. At larger ε_bg_, a larger ϵ_LJ_ is required to maintain the density and surface tension, and *T*_m_ rises sharply. At smaller ε_bg_, the stronger electrostatic interaction may promote dipolar alignment or ordered packing, partially offsetting the benefit of the reduced LJ attraction and producing a modest increase in *T*_m_. Detailed mechanisms of dipolar ordering and its interplay with the LJ attraction during freezing would require further structural analysis, which is beyond the scope of the present parametrization study.

**Figure 8:**
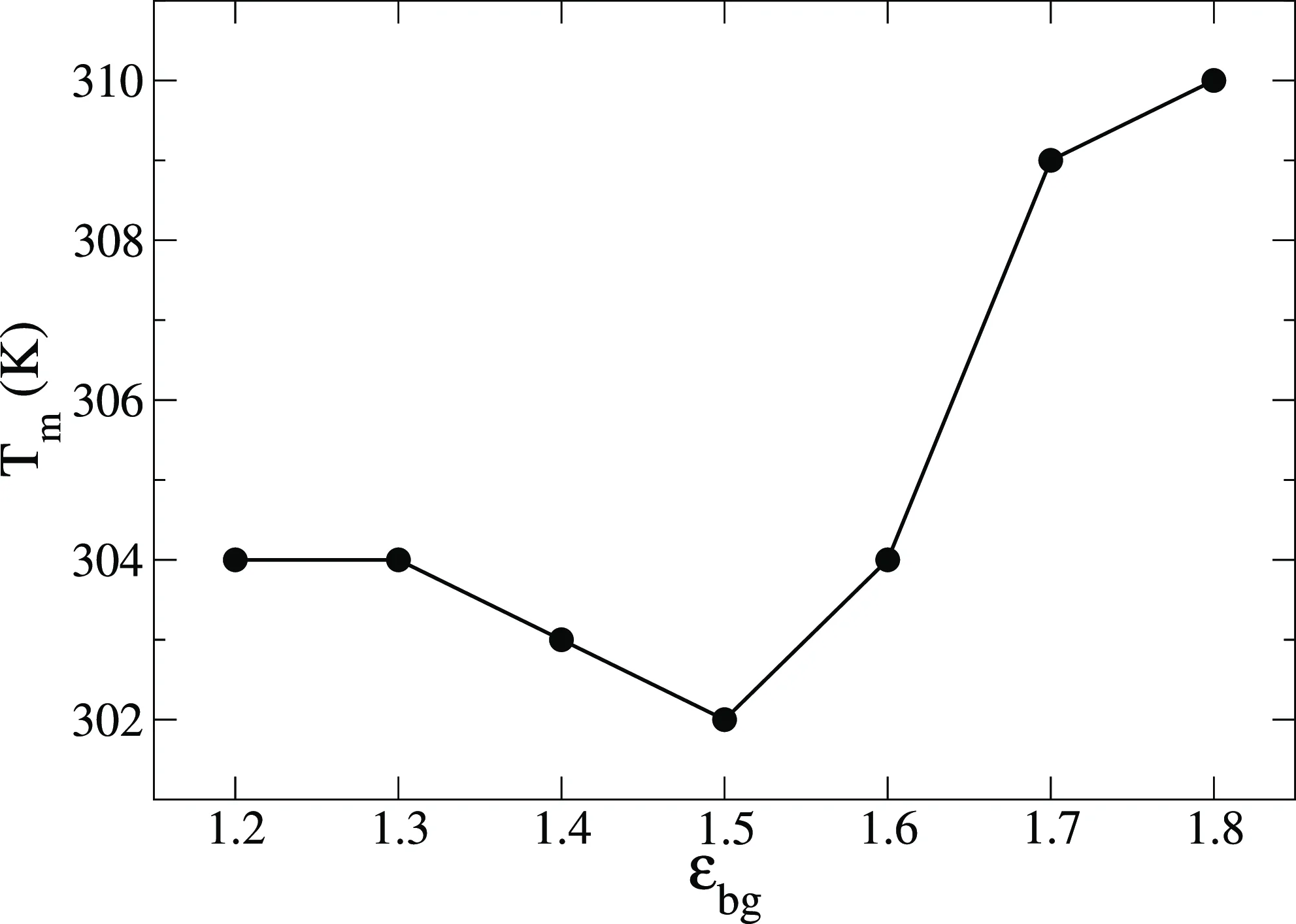
Melting temperature (*T*_m_) as a function of the background dielectric constant ε_bg_. At each ε_bg_, the Lennard–Jones parameters (ϵ_LJ_) and σ_LJ_ were selected using the established parameter-property correlations to reproduce the experimental density and surface tension at 298 K, following the parametrization framework shown in Figure 7.

The asymmetry of the *T*_m_ curve indicates that, along the parametrization path examined, freezing is more sensitive to increases in ϵ_LJ_ than to comparable changes in ε_bg_. We therefore selected ε_bg_ = 1.5 for pSPICA2. At this value, ϵ_LJ_ and σ_LJ_ were further refined to reproduce the density and surface tension at 298 K. Because *r*_0_ altered ε_r_ while having only minor effects on ρ and γ over the examined range, *r*_0_ and the partial charges *Q*_WO_ and *Q*_WH_ were then adjusted at a fixed dipole moment of 4.4 D to reproduce the target dielectric permittivity. The refinement of ϵ_LJ_ and σLJ, followed by adjustment of *r*_0_ and the charges, was iterated until the target properties converged. The final pSPICA2 parameter set is listed in Table 1.

At 298 K, pSPICA2 reproduces the bulk density, surface tension, and dielectric permittivity to within approximately 1% of the experimental values (Table 2). The surface-tension overestimation of the original pSPICA parametrization is largely removed, and the dielectric permittivity remains close to the experimental value. Figure 2 shows that pSPICA2 also retains the overall temperature dependence of ρ­(*T*) over 290–363 K. At the upper end of this range, its density decreases somewhat more steeply than that of pSPICA and deviates more from experiment, possibly reflecting the stronger electrostatic contribution at lower ε_bg_. The γ­(*T*) profile is reproduced well at low and intermediate temperatures. Thus, pSPICA2 preserves the principal temperature-dependent liquid properties of the SPICA family, although some high-temperature deviations remain.

The principal improvement is the substantially lower freezing tendency. The operational *T*_m_ decreases from 384 K for pSPICA to 302 K for pSPICA2, although it remains 29 K above the experimental value and slightly above room temperature. We also performed 50 independent nucleation simulations at 240 K using the same protocol applied to the other models. Only one trajectory froze within the simulation time window, preventing reliable fitting of ΔG*, n*, or *R*_G_ for pSPICA2 (Table 2). Under the identical nucleation protocol, the fraction of trajectories that froze decreased from 50 out of 50 for SPICA, pSPICA, p3SPICA-120, and p3SPICA-104.5 to 1 out of 50 for pSPICA2, indicating a substantial kinetic suppression of freezing.

These results demonstrate that rebalancing the vdW and electrostatic contributions can substantially mitigate the freezing artifact in a polar CG water model while preserving its targeted thermodynamic and dielectric properties. The parametrization logic provides a practical starting point for related models, and its extension to other CG water models requires further testing in the future.

## Conclusions

We systematically examined CG water models of the SPICA FF family with different charge topologies: the uncharged model (SPICA), the two-site linear dipole (pSPICA), and two three-site V-shaped charge distributions (p3SPICA-120 and p3SPICA-104.5). All polar models were parametrized to accurately reproduce the experimental density, surface tension, and dielectric permittivity at 298 K. This shows that an explicit dipole can be introduced and the LJ parameters can be refitted accordingly to match target water properties. Changes in charge topology were therefore necessarily associated with variations in the LJ parameters, and differences in the resulting properties reflected the combined effects of both. We also compared a mean-force-derived effective screening constant ε_eff_ with the dielectric permittivity ε_r_ obtained from equilibrium dipole fluctuations. The two measures were mutually consistent for the parameter sets examined, supporting the use of the fluctuation method as an operational dielectric measure for CG models with an explicit ε_bg_. In addition, varying the dipole length *r*_0_ at fixed dipole moment changed ε_r_ substantially while producing only small changes in density and surface tension over the tested range, providing a useful parametrization degree of freedom.

Despite the comparable ambient properties, the four models exhibited markedly different freezing behaviors. Particularly, under the parametrization constraints of density and surface tension, changing the charge topology from a linear dipole to a V-shaped distribution modified the freezing kinetics: the postcritical growth rate decreased from the nonpolar model to the linear dipole and then to the V-shaped geometries. Yet, changing the charge topology did not eliminate the freezing artifact as all tested polar models retained higher *T*_m_ than nonpolar SPICA. Charge topology therefore affected mainly the kinetics of nucleus growth rather than the melting point itself. In contrast, *T*_m_ across the four models was found to increase monotonically with the parametrized ϵ_LJ_ despite differences in charge topology and LJ functional form. When ϵ_LJ_ was varied in coexistence simulations, all four models showed a liquid-to-crystalline transition within a narrow interval near 0.7–0.9 kcal/mol under the same testing protocol. This indicates that *T*_m_ is strongly sensitive to the LJ interaction strength, regardless of the charge topology or LJ functional form. This motivated us to rebalance the LJ and electrostatic contributions to mitigate the freezing artifact.

On the basis of these results, we proposed a framework that strengthens the explicit electrostatic contribution by reducing ε_bg_ and correspondingly weakens the LJ attraction while preserving target values of density, surface tension, and dielectric permittivity. The resulting pSPICA2 model reduced the operational *T*_m_ from 384 K for pSPICA to 302 K. At 298 K, it retained a density of 1.003 g/cm^3^, a surface tension of 71.9 mN/m, and a dielectric permittivity of 79.1. It also reproduced the principal temperature dependence of density and surface tension over 290–363 K, although deviations remained at elevated temperatures. Under the same 240 K nucleation protocol used for the other models, the fraction of trajectories with freezing events decreased from 50 out of 50 for the four original models down to 1 out of 50 for pSPICA2.

The pSPICA2 model therefore mitigates rather than eliminates the freezing artifact, as its operational *T*_m_ remains above the experimental value of 273 K while the freezing kinetics are substantially suppressed. Nevertheless, the present results show that polarity can be introduced into the SPICA water framework without retaining the full freezing tendency of the original pSPICA model. More broadly, the strategy of rebalancing electrostatic and vdW interactions may provide a useful starting point for developing other explicitly charged CG water models, provided that transferability, interfacial composition, dynamical properties, and compatibility with the surrounding force field are evaluated independently.

## Supplementary Material



## Notes

Test sets of ε_bg_, ϵ_LJ_, σ_LJ_, the corresponding water density ρ and surface tension γ at 298 K, and the resulting linear correlations; the ϵ_LJ_ and σ_LJ_ combinations at various ε_bg_ satisfying experimental ρ = 1 g/cm^3^ and γ = 72 mN/m predicted using the linear correlations (PDF)

## #

Fritz-Haber-Institute of the Max-Planck-Society, 14195 Berlin, Germany
