# Supplementary material for: Mitigating Freezing Artifacts in Polar Coarse-Grained Water through Rebalancing Electrostatic and van der Waals Interactions: A Case Study of the pSPICA Force Field
Source: J Phys Chem B. 2026 Oct 8;130(42):11178–89. doi: 10.1021/acs.jpcb.6c06050 (PMC13699997; doi:10.1021/acs.jpcb.6c06050)
Supplement: Supplementary file 1 [file jp6c06050_si_001.pdf]

Supporting Information

Mitigating Freezing Artifacts in Polar Coarse-Grained Water through Rebalancing Electrostatic and van der Waals Interactions: A Case Study of the pSPICA Force Field

Yi-Chen Tsai,[†,⊥] *Qun-Yan Cheng,*[†] *Wataru Shinoda,*[‡] *and Chi-cheng Chiu*[*,†,¶,§,∥]

† *Department of Chemical Engineering, National Cheng Kung University, Tainan 701, Taiwan*

‡ *Research Institute for Interdisciplinary Science, Okayama University, Okayama 700-8530, Japan*

¶ *Center for Resilience and Intelligence on Sustainable Energy Research (RiSER), National Cheng Kung University, Tainan 701, Taiwan*

§*Program on Smart and Sustainable Manufacturing, Academy of Innovative Semiconductor and Sustainable Manufacturing, National Cheng Kung University, Tainan 701, Taiwan*

∥*Fire Protection and Safety Research Center, National Cheng Kung University, Tainan 711, Taiwan*

⊥ *Current address: Fritz-Haber-Institute of the Max-Planck-Society, 14195 Berlin, Germany*

[*] *Corresponding Authors, E-mail: ccchiu2@gs.ncku.edu.tw*

1. Parameterization of pSPICA2

Following the parameterization framework outlined in Fig. 7 of the main text, we first explored the LJ 12-5 potential form for the WO–WO vdW interaction, consistent with the original pSPICA parameterization. We tested three representative background dielectric constants (ε_{bg} = 1.6, 2.0, and 3.2), each sampled with a set of ϵ_{LJ} and σ_{LJ} values. The resulting bulk density ρ and surface tension γ at 298 K were obtained from independent simulations as listed in Table S1.

Table S1. Test sets of ε_{bg}, ϵ_{LJ} , and σ_{LJ} using LJ12-5 potentials and the corresponding water density ρ and surface tension γ at 298 K for establishing correlations.

ε_{bg}	ϵ_{LJ} (kcal/mol)	σ_{LJ} (Å)	ρ (g/cm^3)	γ (mN/m)
1.6	0.65	4.236	1.138	55.67
		4.324	1.058	50.30
		4.412	0.984	45.98
	0.70	4.236	1.148	61.16
		4.324	1.067	55.70
		4.412	0.994	51.32
	0.75	4.236	1.156	66.42
		4.324	1.031	60.92
		4.412	0.959	55.43
2.0	0.65	4.236	1.109	55.73
		4.324	1.031	50.93
		4.412	0.959	46.91
	0.70	4.236	1.120	61.33
		4.324	1.042	56.03
		4.412	0.971	51.58
	0.75	4.236	1.131	61.33
		4.324	1.053	56.03
		4.412	0.981	51.58
3.2	0.65	4.236	1.037	38.61
		4.324	0.966	35.31
		4.412	0.900	32.00
	0.70	4.236	1.055	44.23
		4.324	0.983	40.08
		4.412	0.918	36.43
	0.75	4.236	1.071	49.49
		4.324	0.998	45.58
		4.412	0.933	41.57

Linear regressions were performed to establish the correlations between ρ, γ, and the non-bonded parameters ε_{bg}, ϵ_{LJ} , and σ_{LJ}:

$$\rho\left(\varepsilon_{\mathrm{bg}}, \epsilon_{\mathrm{LJ}}, \sigma_{\mathrm{LJ}}\right) = 4.58058 - 0.05213\varepsilon_{\mathrm{bg}} + 0.24184\epsilon_{\mathrm{LJ}}, -0.83234\sigma_{\mathrm{LJ}} , \qquad R^2 = 0.996$$

$$\gamma\left(\varepsilon_{\mathrm{bg}}, \epsilon_{\mathrm{LJ}}, \sigma_{\mathrm{LJ}}\right) = 216.40248 - 9.53362\varepsilon_{\mathrm{bg}} + 102.86656\epsilon_{\mathrm{LJ}}, -50.33965\sigma_{\mathrm{LJ}}, \qquad R^2 = 0.994$$

The corresponding response surfaces for the water density ρ and surface tension γ, together with the test data sets, are shown in Fig. S1 and Fig. S2, respectively.

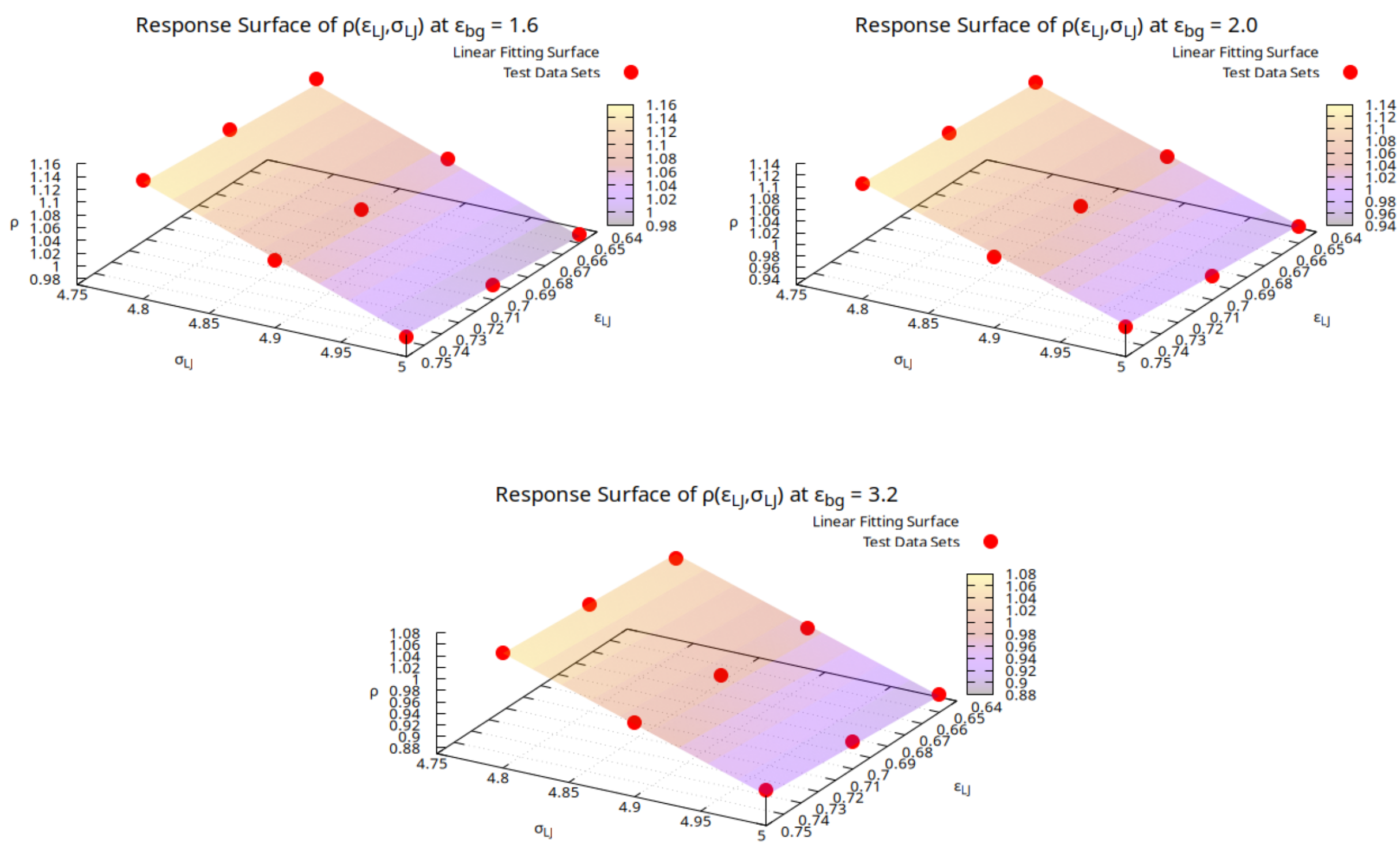

Fig. S1 Response surface of $\rho\left(\epsilon_{\mathrm{LJ}}, \sigma_{\mathrm{LJ}}\right)$ with $\varepsilon_{\mathrm{bg}} = 1.6, 2.0, 3.2$ at 298 K along with test data sets obtained from simulations.

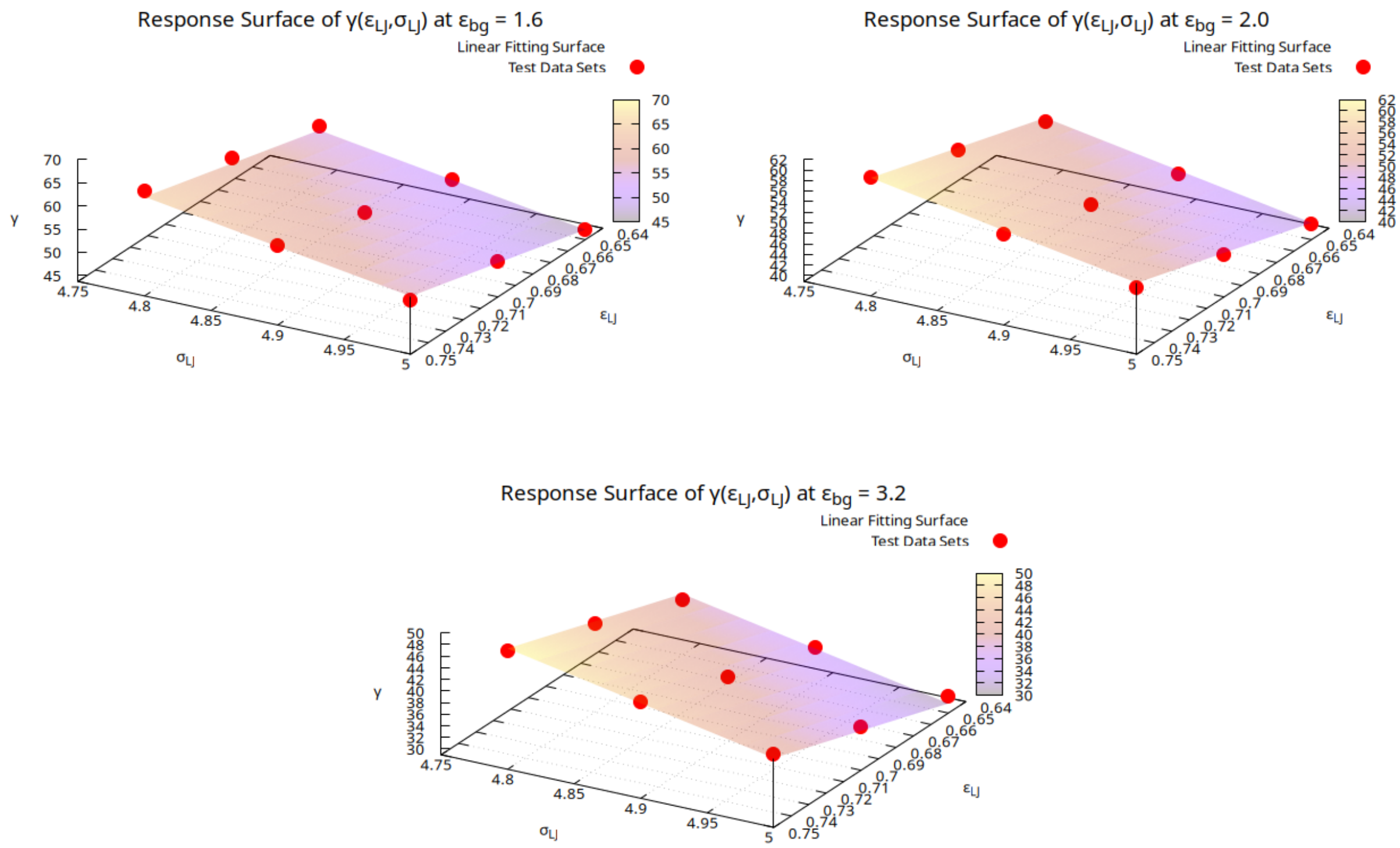

Fig. S2 Response surface of $\gamma\left(\epsilon_{\mathrm{LJ}}, \sigma_{\mathrm{LJ}}\right)$ with $\varepsilon_{\mathrm{bg}} = 1.6, 2.0, 3.2$ at 298 K along with test data sets obtained from simulations.

Using these correlations, however, we found that the LJ 12-5 form could not simultaneously satisfy the experimental ρ = 1 g/cm^3 and γ = 72 mN/m once ϵ_{LJ} was reduced below approximately 0.7 kcal/mol, the regime required to suppress the freezing artifact as established in Fig. 6 in the main text. This limitation motivated us to switch to the LJ 12-4 potential form, which was originally adopted in the non-polar SPICA water model and offers a longer attractive range to accommodate the high γ of water.

The same parameterization procedure was repeated using the LJ 12-4 potential. Test sets of ε_{bg}, ϵ_{LJ} , and σ_{LJ}, along with the corresponding ρ and γ at 298 K, are summarized in Table S2. The resulting linear correlations are:

$$\rho\left(\varepsilon_{\mathrm{bg}}, \epsilon_{\mathrm{LJ}}, \sigma_{\mathrm{LJ}}\right) = 5.15433 - 0.07396\varepsilon_{\mathrm{bg}} + 0.16379\epsilon_{\mathrm{LJ}}, -0.93011\sigma_{\mathrm{LJ}}\,, \qquad R^2 = 0.998$$

$$\gamma\left(\varepsilon_{\mathrm{bg}}, \epsilon_{\mathrm{LJ}}, \sigma_{\mathrm{LJ}}\right) = 390.86429 - 18.36331\varepsilon_{\mathrm{bg}} + 145.75843\epsilon_{\mathrm{LJ}}, -88.02770\sigma_{\mathrm{LJ}}, \qquad R^2 = 0.995$$

Table S2. Test sets of ε_{bg}, ϵ_{LJ} , and σ_{LJ} using LJ 12-4 potentials and the corresponding water density ρ and surface tension γ at 298 K for establishing correlations.

ε_{bg}	ϵ_{LJ} (kcal/mol)	σ_{LJ} (Å)	ρ (g/cm^3)	γ (mN/m)
1.6	0.65	4.184	1.258	88.31
		4.271	1.168	79.54
		4.358	1.086	72.15
	0.70	4.184	1.265	95.92
		4.271	1.176	87.13
		4.358	1.094	79.68
	0.75	4.184	1.271	104.07
		4.271	1.182	94.95
		4.358	1.101	85.97
2.0	0.65	4.184	1.221	79.81
		4.271	1.134	72.35
		4.358	1.055	65.13
	0.70	4.184	1.231	88.16
		4.271	1.144	79.46
		4.358	1.065	71.99
	0.75	4.184	1.239	95.92
		4.271	1.153	86.92
		4.358	1.074	78.56

Unlike the LJ 12-5 form, the LJ 12-4 correlations remained valid across the full ϵ_{LJ} range required to satisfy both ρ = 1 g/cm^3 and γ = 72 mN/m, including the low-ϵ_{LJ} regime relevant for freezing suppression. The LJ 12-4 form was therefore adopted for the subsequent development of pSPICA2.

Using the LJ 12-4 correlations above, ϵ_{LJ} and σ_{LJ} were predicted for ε_{bg} ranging from 1.2 to 1.8, under the constraints of ρ = 1 g/cm^3 and γ = 72 mN/m at 298 K as listed in Table S3. These predicted parameter sets were used to perform the coexistence simulations shown in Fig. 8, from which ε_{bg} = 1.5 was selected as the optimal value. Further refinement of ϵ_{LJ} and σ_{LJ}, together with adjustment of the dipole geometry (r_0 and partial charges) at fixed dipole moment, yielded the final pSPICA2 parameter set reported in Table 1 in the main text.

Table S3. Estimated ϵ_{LJ} and σ_{LJ} using LJ 12-4 potentials for ε_{bg} = 1.2 ~ 1.8 and using the fitted correlations and the constraints of ρ = 1 g/cm^3 and γ = 72 mN/m at 298 K.

ε_{bg}	ϵ_{LJ} (kcal/mol)	σ_{LJ} (Å)
1.2	0.675	4.490
1.3	0.684	4.484
1.4	0.693	4.477
1.5	0.701	4.471
1.6	0.710	4.465
1.7	0.719	4.458
1.8	0.728	4.452
